# A Case of Rhinolith and Two Cases of a Tooth in the Nose

**Published:** 1889-10

**Authors:** Jonathan Wright

**Affiliations:** Brooklyn, N. Y.


THE
AMERICAN JOURNAL
—OF—
DENTAL SCIENCE.

VOL. XXIII. BALTIMORE, OCTOBER, 1889. No. 6.

ARTICLE I.

### A CASE OF RHINOLITH AND TWO CASES OF A TOOTH IN THE NOSE.

BY JONATHAN WRIGHT, M. D., BROOKLYN, N. Y.

While rhinoliths are by no means very frequently found, yet they are not so rare as to be unknown in the experience of any laryngologist who sees annually a large number of cases of nasal disease. They are more often seen in dispensary practice than among private patients. This, no doubt, is due to the neglect of symptoms caused by the presence of a foreign body in the nose among this class, and to the fact that the children of well-to-do parents are more carefully watched, and the introduction of foreign bodies prevented or detected. A rhinolith always has as a nucleus a foreign body which, in the great majority of cases, whatever the age of the patient, has been introduced into the nose during childhood, although it is the exception to obtain a history of the event. After the lapse of time it gradually becomes coated with lime-salts, deposited upon it by the nasal secretions, rendered abnormally abundant by the irritation of its presence. The longer it remains the larger it grows, and the greater the local irritation it causes. The nucleus is most frequently a relatively small object, because a large one soon causes such symptoms as to be noticed by the parents of the child, or by the patient himself, and thus becomes the subject of investigation. The nucleus may be anything, and nearly everything has been found, from shoe-buttons and pieces of coal, to maggots and parasites. The rhinolith, as it grows by the accretion of lime-salts, hollows out for itself a cavity in the nasal fossa, usually the inferior meatus, and this cavity may be of considerable size. The bony walls may be encroached upon, causing necrosis of bone, but the destructive process is rarely, if ever, extensive. After a time, a condition of the nose exists which, at first glance, has every appearance of idiopathic ozæna. There may even be atrophy of the mucous membrane. This appearance is always unilateral, while ordinary ozæna rarely, though occasionally, is. A frequent symptom is excessive epistaxis, especially when the nasal fossa is probed. The foul smell, which may be that of necrosed bone, often resembles syphilitic ozæna. Pain of varying intensity may be present, as well as various reflexes, such as sneezing, coughing, asthma, neuralgias—in short, any or all the symptoms due to intra-nasal pressure, or to irritation from other causes.

The nasal discharge is usually copious, and may be thick, forming crusts easily, or muco-purulent, stained with blood, or simply watery. Occasionally portions of the lime salts may come away with the discharges, the patient speaking of them as "gritty." Such symptoms, when unilateral, are almost pathognomonic of a foreign body in the nose, which may or may not have lime-salts around it.

A rhinoscopic examination reveals, as a rule, no foreign body to the view, because it is imbedded in its cavity and surrounded by hypertrophic mucous membrane; cleansing the nasal fossæ by a spray fails to bring it to sight. The application of cocaine, by shrinking the tissues and enlarging the field of vision, may often disclose the source of the trouble. The probe is the most reliable means of diagnosis, the grating of the hard body revealing its presence. The presence of dead bone from syphilitic necrosis is practically the only other condition which can deceive the experienced examiner. This can still further be eliminated by measuring with the bent probe the limits and the shape of the bared surface, and by its mobility.

If the foreign body is not easily extracted at the first sitting, it is well to give the patient an alkaline nose-wash with a syringe, and direct him to keep the nose clean for a few days by the use of them. Often, at the next visit the discharges and the swelling, as well as the subjective symptoms, will have largely abated. A stiff probe is often useful in prying the body out of its cavity. By bending a right-angled hook on the end of the probe the body may be dragged out by this simple means. A snare may be used to encircle it, washing the nose out from behind with a post-nasal syringe; a sudden expiratory effort by the patient, after the object has been disloged, may bring it out. The best forceps for the purpose are those devised for general nasal work by Dr. C. H. Knight, of New York City. I believe there have been various instruments specially devised for extracting a foreign body from the nose. I doubt if they are necessary.

Of late years, the journals devoted to laryngology and rhinology have abounded in descriptions of cases of rhinoliths and other foreign bodies in the nose, and a reference to them will easily disclose the literature of the subject. The case of rhinolith reported here is one of several that have come under my observation, distinguished from others simply by its size and the character of the nucleus.

D. L——, aged thirteen, a schoolboy, applied at the Demilt Dispensary for treatment on April 27, 1888. Three years previously his nose had begun to trouble him, bleeding easily and discharging offensive material, and always from the left side. No history could be obtained of the introduction of any foreign body whatever.

There was marked tenderness to touch on the left side of the nose. An intra-nasal examination showed an object covered with secretions lying in the left inferior meatus. A foul odor was emitted from this side of the nose. The object seemed about the size of a hazel-nut, and gave a grating sound and sensation on the impingement of a probe. It was necessary to break it into several fragments before extraction. It was found to consist of two layers of hard calcareous material, between which sponge-tissue could be plainly distinguished. The calcareous material had infiltrated only the outer portion of the sponge, leaving the central part free from it. The rhinolith had hollowed out for itself a nest between the septum, the inferior turbinated bone, and the floor of the inferior meatus; all nasal symptoms soon subsided after the extraction.

As has been said, many such cases have been reported. The presence of a tooth in the nose is, however, a very rare circumstance. There is a record of one case, where a right upper canine tooth was removed from the left orbit of a child. This was evidently a dentigerous cyst, which must have begun development at a very early stage of embryonic life. Many cases of dentigerous cysts have been reported, and they are not by any means confined to the maxillary regions.

Dr. Wyeth, several years ago, reported to the New York Pathological Society a case in which a displaced tooth was found in the antrum of Highmore. Dr. Griffin found a tooth growing upward in the nose in a case of cleft palate. This produced no symptoms. Dr. Marshall, and Dr. Ingalls, of Chicago, have each reported a case in which the tooth had apparently ulcerated through into the nose, from the upper jaw. It is doubtful how the teeth in the cases reported by Schaeffer, Roy, and Hall became nasal inhabitants. These six cases are all that I have been able to find recorded, after a careful search, though other reports may have been overlooked. In all the cases reported the teeth were the anterior ones, canines or incisors; and the reasons are obvious, since the posterior ones, if inverted, as in Griffin's case, would grow into the antrum, and probably produce no symptoms. I have seen skulls in which a tooth had attained full growth within the alveolar process of the superior maxilla without emerging from the bone at any point. Where ulceration takes place, the progress of a bicuspid or tricuspid upward would be very unlikely to occur, from the shape of its root. If it should occur, as in the case of Wyeth, it would, from its situation, be most likely to be discharged into the antrum.

The first of the cases related here is instructive as illustrating the migration of an object through bony structure, while the second case was evidently originally one of the extra-alveolar development.

Case I.—Mary V——, aged thirty-five; married; came to the Demilt Dispensary February 21, 1888. Her previous and family histories had no bearing on the case. Four years previously she had all her remaining teeth, nine in number, extracted from the upper jaw. The last one, a lateral incisor or canine, on the left side, she had extracted during nitrous oxide narcosis. The dentist told her he thought he had left a piece behind, but could not detect it on probing. There was a good deal of gingival tenderness afterward, which soon subsided. These facts were elicited from direct questioning after the nature of the trouble became evident, the patient having had no suspicion of the connection between the tooth episode and the nasal symptoms which followed six months later. At that time the left side of her nose began to trouble her, by being "stopped up" at times, and there was a slight discharge. For two years previous to her application for treatment there had occasionally been a good deal of bleeding, and the obstruction, together with the nasal discharge, had steadily increased. For the last six months the nose on the left side had been painful and sensitive. An intra-nasal examination showed marked hypertrophy of the nasal mucous membrane on the left side. It was red and inflamed, add bled easily.

A probe detected a rough, hard surface about an inch and a half from the left anterior meatus, on the floor of the nose and close to the septum. Small pieces of calcareous matter or bone could be crumbled off with the probe. The patient was given an alkaline wash with a syringe. Two weeks later she again presented herself, bringing with her a small, flat sequestrum which she had washed out of her nose. After the thorough application of cocaine, by means of a probe and the forceps the single root of a tooth, about three-fourths of an inch long, was extracted from the left nostril. At one end there was some calcareous matter firmly adherent. The nasal symptoms soon subsided.

CASE II.—Mary F——, aged eight, applied for treatment to the Throat Class of the Roosevelt Dispensary on August 17, 1888. Her father died of phthisis. The child was undersized, and had evidentally been a sufferer from rachitis. The mother could give little or no previous history of the case, except that the child's nose, as far as she knew, was in a normal condition when she was a year old. Since then the nose had been stopped up so as to prevent nasal breathing. There had been considerable offensive discharge. The bridge of the nose was sunken. The nostrils were flattened and contracted. The superior alveolar process on the left side, corresponding to the situation of the incisor and canine teeth, was absent, leaving a slight cleft. No history of accident could be obtained, the mother stating that she had never noticed the defect before. The left nostril was obstructed by a foreign body, which gave a grating sound on the impingement of a probe. Considerable force was necessary to extract it with the forceps. It was found to be the crown of an incisor tooth. It was notched, as in young children, and larger than in a child a year old. There was considerable calcareous matter around it and in the nostril, but no root was found.

The patient's nasal symptoms had very markedly abated at the next visit, and since then she has not been seen.—*Medical Record.*

